# Neuromodulation Effect of Very Low Intensity Transcranial Ultrasound Stimulation on Multiple Nuclei in Rat Brain

**DOI:** 10.3389/fnagi.2021.656430

**Published:** 2021-04-15

**Authors:** Yingjian Liu, Gang Wang, Chao Cao, Gaorui Zhang, Emily B. Tanzi, Yang Zhang, Weidong Zhou, Yi Li

**Affiliations:** ^1^School of Microelectronics, Shandong University, Jinan, China; ^2^Department of Radiology, Qilu Hospital of Shandong University, Jinan, China; ^3^School of Medical Imaging, Weifang Medical University, Weifang, China; ^4^Weill Cornell Medicine, New York, NY, United States

**Keywords:** transcranial ultrasound stimulation, local field potentials, neuromodulation, hippocampus, ephaptic coupling, neuronal resonance, synchronization

## Abstract

**Objective:**

Low-intensity transcranial ultrasound stimulation (TUS) is a non-invasive neuromodulation technique with high spatial resolution and feasible penetration depth. To date, the mechanisms of TUS modulated neural oscillations are not fully understood. This study designed a very low acoustic intensity (AI) TUS system that produces considerably reduced AI Ultrasound pulses (*I*_*SPTA*_ < 0.5 W/cm2) when compared to previous methods used to measure regional neural oscillation patterns under different TUS parameters.

**Methods:**

We recorded the local field potential (LFP) of five brain nuclei under TUS with three groups of simulating parameters. Spectrum estimation, time-frequency analysis (TFA), and relative power analysis methods have been applied to investigate neural oscillation patterns under different stimulation parameters.

**Results:**

Under PRF, 500 Hz and 1 kHz TUS, high-amplitude LFP activity with the auto-rhythmic pattern appeared in selected nuclei when I_*SPTA*_ exceeded 12 mW/cm^2^. With TFA, high-frequency energy (slow gamma and high gamma) was significantly increased during the auto-rhythmic patterns. We observed an initial plateau in nuclei response when I_*SPTA*_ reached 16.4 mW/cm^2^ for RPF 500 Hz and 20.8 mW/cm^2^ for RPF 1 kHz. The number of responding nuclei started decreasing while I_*SPTA*_ continued increasing. Under 1.5 kHz TUS, no auto-rhythmic patterns have been observed, but slow frequency power was increased during TUS. TUS inhibited most of the frequency band and generated obvious slow waves (theta and delta band) when stimulated at RPF = 1.5 kHz, *I*_*SPTA*_ = 8.8 mW/cm^2^.

**Conclusion:**

These results demonstrate that very low intensity Transcranial Ultrasound Stimulation (VLTUS) exerts significant neuromodulator effects under specific parameters in rat models and may be a valid tool to study neuronal physiology.

## Introduction

Low-intensity transcranial ultrasound stimulation (TUS) is a non-invasive neuromodulation technique that can be reliably transmitted through the skull to stimulate neurons with high spatial resolution and deep penetration depth (Hayner and Hynynen, 2001). TUS could change behavioral and electrophysiological processes by stimulating particular regions of the brain, (Yoo et al., 2011) and it has been proved an effective tool in clinical neurosciences (Tufail et al., 2010). Recent studies show that TUS has much neurotherapeutic potential in ischemic brain injury, (Guo et al., 2015) epilepsy, (Hakimova et al., 2015) depression, (Zhang et al., 2018), and Alzheimer disease (Lin et al., 2015).

Neuromodulation has been implemented broadly in neuroscience research, with several methods developed in recent years. Electrical methods such as deep-brain stimulation (DBS) have a high targeting spatial resolution and are considered highly effective therapies for disease intervention. But DBS suffers limitations such as invasiveness, and brain impalement with electrodes. Transcranial magnetic stimulation (Barker, 1999; Perera et al., 2016) does not require surgery but suffers from low spatial resolution. Optogenetic-based approaches have extraordinary spatial precision but require genetic manipulation. These kinds of methods have inevitably limited applications. In addition to the aforementioned methods, TUS is a promising non-invasive neuromodulation technology with better spatial resolution and higher penetration depth (Hayner and Hynynen, 2001). Many studies were conducted to measure the effects of ultrasound stimulation on neural oscillations *ex vivo* or *in vivo* (Bystritsky et al., 2011). In contrast to high intensity US, low-intensity US (*I*_*SPPA*_: 0.5–100 W/cm^2^), is delivered in a pulsed mode for brief periods of time, has been widely used in animal neuromodulation studies and can directly stimulate action potentials and synaptic transmission through mechanisms involving the non-thermal activation of ion channels (Tyler et al., 2008).

Local field potential (LFP) recorded extremely local phenomena within the nuclei, so it can accurately measure the brain function changes after ultrasonic stimulation. Previous studies have recorded LFPs in the motor cortex before and after ultrasound stimulation with different stimulation parameters (Wang et al., 2019), and demonstrates that TUS can significantly decrease parkinsonian-related activity in mice administered MPTP (Wang et al., 2020). By using LFPs to analyze the changes quantitatively, Yuan pointed out that neural activities and hemodynamic response of the mouse visual cortex induced by TUS are related to the ultrasound intensity (Yuan et al., 2021). LPF can also provide electrophysiological recording, which proves that cortical hemodynamics changes induced by TUS are related to stimulation intensity and duration (Yuan et al., 2020). Therefore, we investigate the change of electrophysiological responses across different brain regions under different TUS parameters by using LPFs.

Ultrasound directly activates a localized area, (Tufail et al., 2010) or indirectly activates auditory pathways that, in turn activate other cortical networks (Guo et al., 2018; Sato et al., 2018). Another study also showed TUS induced hemodynamic responses; however, the acoustic intensity (AI) was much higher in that study than the Food and Drug Administration (FDA) guidelines defined (*I*_*SPTA*_ ≤ 94 mW/cm^2^) (Center for Devices and Radiological Health, 2019). The neuronal membrane is a dedicated structure, different neuronal oscillation pattern may occur under the very low intensity US simulation. Here we designed a very low AI TUS system that can produce much lower AI pulse (*I*_*SPPA*_ < 0.5 W/cm^2^, *I*_*SPTA*_ < 100 mW/cm^2^) to investigate the minimum stimulus intensity of nerve oscillation in different brain regions. What’s more, we hope to reduce power consumption of ultrasonic neuromodulation and to reduce retroinhibition by high AI TUS.

Despite the broad application potential of ultrasound, the multi-nucleus mechanisms underlying TUS-modulated neural oscillations remain poorly understood. Most ultrasonic neural regulation is limited to the study of LFP in a single nucleus. In this study, we hope to explore the differences of nuclei under ultrasound stimulation from the perspective of multiple nuclei. Five nuclei, SI, CA1, CA2, LHb, and MI, have been selected in this study to examine different brain networks crossing motor, sensory, perception, and cognition (Rinaldi et al., 1991; Bachtold et al., 1998; Matsumoto and Hikosaka, 2009). These five nuclei are also associated with memory and feeling. Therefore, understanding the response of these nuclei after ultrasonic stimulation can provide a significant value for the ultrasound treatment of many neurological diseases.

In this study, we designed a very low-intensity TUS system (*I*_*SPTA*_ < 30 mW^2^) to stimulate the rat brain under different frequencies and AIs. LFP signals of five nuclei were recorded simultaneously and processed by spectrum estimation, time-frequency analysis (TFA), and relative power analysis methods with three sets of parameters at different ultrasonic intensities to investigate the TUS induced neuromodulation. To the best of our knowledge, this study is the first to analyze neuromodulation effects of deep brain nuclei under very low intensity transcranial ultrasound stimulation (VLTUS).

## Materials and Methods

### Animal Anesthesia and Surgery

**FIGURE 1:**
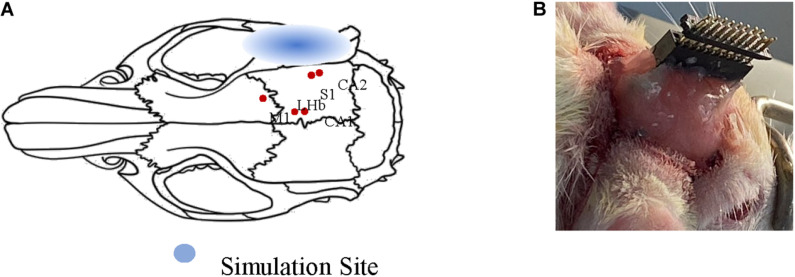
Schematic diagram of animal surgery. **(A)** Anatomy map of rat. The relative positions of the five nuclei are marked with red dots. **(B)** The final form of surgery. The electrodes are sealed only the interface of electrodes were exposed.

Two adult Sprague–Dawley rats (male: 270–310 g) were purchased from the Laboratory of Animal Center of Zhejiang University. Both rats were anesthetized with chloral hydrate solution (10%, 0.35 ml/100 g). The anesthetized rats were fixed on a stereotaxic apparatus (51603U, RWD, China) with ear bars and a clamping device to keep their heads horizontal. Specifically, the fur covering each rat’s skull was trimmed to expose the cleaner scalp, and the scalp was sterilized with iodine. The bregma and lambda were explored after cutting the scalp and removing the subcutaneous tissue. One hole was drilled into the rats’ meninges to fix reference electrodes. Another hole was drilled at the intersection of the sagittal suture and the interaural line as the ground. Five recorded sites were determined according to the reference (Paxinos and Watson, 2006) (primary sensory cortex, SI: AP = −4.44 mm, ML = 6 mm, DV = 3 mm; primary motor cortex, MI: AP = 0.48, ML = 2.2, DV = 2; lateral habenular nu, LHb: AP = −2.52, ML = 0.6, DV = 4.8; field CA1 of the hippocampus, CA1: AP = −3.96, ML = 1.2, DV = 3.4; field CA2 of the hippocampus, CA2: AP = −5.20, ML = 5.6, DV = 6). Figure 1A shows the anatomy map of the rat. It visualizes the sites of LFP recorded under different ultrasound stimulation parameters. The electrode and the head wound of the rat were sealed with dental cement, and only the interface of the recording electrodes was exposed finally. After the animal surgery, the two rats were housed in cages for a week to recover with free access to food and water. This study’s protocols were approved by the Laboratory Animal Ethical and Welfare Committee of Shandong University Cheeloo College of Medicine.

In order to rule out accidental circumstances, we repeated the experiment three times for each stimulus parameter on two adult Sprague–Dawley rats.

### TUS Experimental Setup

**FIGURE 2:**
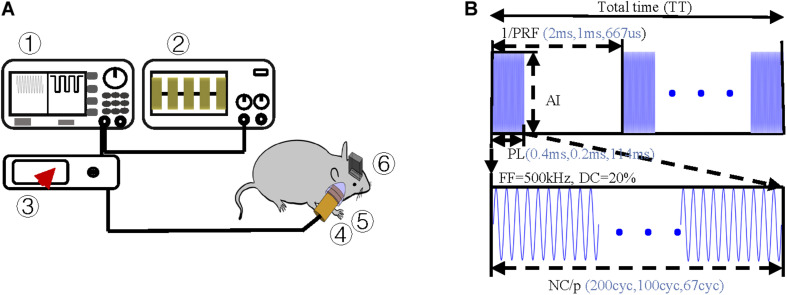
Experimental protocol **(A)** Schematic of ultrasound stimulation and LFP recordings ➀ arbitrary function generator, ➁ oscilloscope, ➂ amplifier, ➃ ultrasound transducer, ➄ acoustic collimator, and ➅ electrodes. **(B)** Schematic of ultrasound sequences and associated parameters, i.e., simulation duration (SD), pulsed repetition frequency (PRF), acoustic intensity (AI), fundamental frequency (FF), and number of cycles per pulse (NC/p).

In the TUS system, an arbitrary function generator (YB16051A, Lv yang, China) was used to control the pulsed repetition frequency (PRF), fundamental frequency (FF), number of cycles per pulse (NC/p), and duty cycle (DC) of ultrasound. The pulsed sequence from the generator was amplified by a high voltage amplifier (ATA-2021H, Aigtek, China) and transmitted to an ultrasound transducer (focal length of 45 mm and focus diameter of 20 mm). The ultrasound transducer was connected to the rat skull by a conical acoustic collimator filled with ultrasound coupling gel (Figure 2A). The collimator is a circular area with a radius of 0.4 cm, so the focus area does not exceed 0.51 cm^2^. The transducer connected to the collimator is 8 mm higher than the skull, so the focal length into the brain is less than 37 mm. As illustrated in Figure 1A, in order to reduce ultrasonic attenuation, electrodes were implanted into the nucleus vertically, and the collimator was aimed horizontally from the occipital side. The angle between them was 90°. The electrode and the head skull of the rat were all sealed with dental cement. In order to avoid the obstruction and attenuation of ultrasound by dental cement, we applied ultrasound to the occipital lobe. The stimulation site was chosen as shown in Figure 1A, 6.5 mm lateral of midline and 4 mm posterior of bregma.

Pulsed ultrasound sequences were constructed as illustrated in Figure 2B. The FF and DC of the ultrasound were 500 kHz and 20% respectively. By using the collimator, the ultrasonic focal spot radius was fixed at 0.4 cm. Under the condition of fixed FF and DC, we designed three groups of trials by changing PRF and NC/p. For each trial, we changed the magnification of the amplifier to get the increasing AI. Three PRFs (500 Hz, 1,000 Hz, and 1,500) were used in this study. The range of I_*SPTA*_ was from 7.2 to 25.6 mW/cm^2^. The ultrasound parameters, spatial peak temporal average intensity (I_*SPPA*_), spatial peak pulse average (I_*SPTA*_), PRF, and NC/p we used are listed in Table 1.

**TABLE 1:** Number of nuclei showed auto-rhythm during VLTUS.

**I_*SPPA*_ (W/cm^2^)**	**I_*SPTA*_ (mW/cm^2^)**	**PRF = 500 HzNC/p = 200**	**PRF = 1 kHzNC/p = 100**	**PRF = 1.5 kHzNC/p = 67**
**0.036**	**7.2**	0	0	0
**0.044**	**8.8**	0	0	0
**0.06**	**12**	1	2	0
**0.082**	**16.4**	**5**	3	0

*I_*SPPA*_: the spatial peak, pulse average intensity; I_*SPTA*_: spatial peak temporal average intensity; PRF: pulse repetition frequency; NC/p: number of cycles per pulse.*

The ultrasonic power generated by the plane transducer (installed on the collimator) was measured by the radiation force method using the ultrasound power meters model (UPM-DT-1AV, Ohmic, United States). During this process, the transducer was held above the de-gassed water by a positioning clamp. The ultrasonic energy passed through the water to reflect off the conical target and was then absorbed by the rubber lining. The radiant power was proportional to the total downward force in the conical target. This weight was then transferred to the electro-mechanical load cell, which could produce a digital readout in watts of power. In this experiment we assumed that the brain and tissues had the sound, speed, and density of water. The final acoustic attenuation transmitted into the brain was 83.9% because of the absorption and refraction properties of the ultrasound in the rat skull. Since the five nuclei are not far apart, the ultrasound intensity at different nuclei is the same. Thus, we only consider the attenuation of a skull. Details of the AI calculation are described in the Appendix.

### Data Acquisition

Five electrodes (nickel-chromium alloy, 35 μm, A-M SYSTEM, United States) were used to record the LFP signals in the designated brain area of rats. Each electrode was made of two nickel-chrome wires that were intertwined with each other. The reference electrode implanted into the rats’ meninges was made in the same way as the recording electrodes. The ground electrode was made of exposed silver wire. Finally, all electrodes were connected to a 16-channel (2 × 8 array) interface.

The raw LFP signals produced in response to TUS were collected by a 32-channel neural signal processor (RHD2132, Intan, United States), which can perform pre-amplification and AD conversion at the same time. These signals were acquired at the sampling frequency of 1 kHz and stored on field programmable gate array (FPGA). Finally, all data were transmitted to the computer and processed using MATLAB 2018b (MathWorks, Inc., United States).

After the final recording session, the animal was perfused with paraformaldehyde (PFA) 4%, and histological analysis was performed to confirm electrode locations. Correct electrode position was confirmed for all animals in this study.

### Spectral Estimation and Time-Frequency Analysis

Spectral estimation can transform a signal from the time domain to the frequency domain and provide a description of the signal’s power distribution along with frequency (Hu and Zhang, 2019). The foundation of spectral estimation is the discrete Fourier transform (DFT), which is calculated using the Fast Fourier transform (FFT) algorithm for computational efficiency. Taking discrete-time signal*x*_*n*_, *n* = 1, 2…N as an example, the DFT of*x*_*n*_can be written as

(1) $$F\,\left(k\right)=\sum_{n=0}^{N-1}x_{n}\,e^{-j\,2\,\pi \,k\,n/N}$$

Based on DFT, the power spectral density (PSD) can be calculated to describe how the power of signal is distributed over different frequencies. In our study, LFP signals were acquired at the sampling frequency of 1 kHz with software designed Bandpass (1–140 Hz, 1–45 Hz) and notch (50, 100 Hz) filter. And LFP powers were computed for individual channels using the multitaper spectral estimation technique (Babadi and Brown, 2014). These powers were then normalized by mean power for the global spectrum (1–140 Hz) within each time serial.

To observe the time-variant spectral components of LFP signals over whole periods, TFA techniques are needed to study such non-stationary signals in both the time and frequency domains simultaneously. The S-transform expresses the phase information by using the Fourier kernel, and translates the amplitude envelope of the Gaussian window. The S-transform of a time-varying LFP signal x(t) is defined as:

(2) $$S_{x}\,\left(t,f\right)=\frac{\left|f\right|}{\sqrt{2\,\pi }}\,\int_{-\infty }^{\infty }x\,\left(\tau \right)\,e^{\frac{-{\left(\tau -t\right)}^{2}\,f^{2}}{2}}\,e^{-i\,2\,\pi \,f\,\tau }\,d\tau$$

### Relative Power Analysis of Different Rhythms

The relative power represented a key power of neuromodulation parameter (Henrie and Shapley, 2005). The upper frequency cutoff of the LFP is often considered to be around 200 Hz. In our study, after spectrum estimation of LFP signals, signals were decomposed into the delta (1–3 Hz), theta (4–8 Hz), alpha (8–12 Hz), beta (12–30 Hz), slow gamma (30–60 Hz), high gamma (60–200 Hz) frequency bands by the discrete wavelet transform (DWT) according to the rhythms (Buzsaki, 2006). Relative power was used to eliminate the difference between experiments. The relative power of each rhythm is equal to the corresponding band-limited power corrected by the global spectrum (1–200 Hz) power. For example, the band-limited power of the alpha rhythm is calculated as the spectral estimation averaged from all frequency points within the range of 8–12 Hz, and the relative power is the ratio between the alpha power and the total power of the LFP signal.

## Results

### Neuromodulation Effect of Five Nuclei Under Different VLUTS Parameters

**FIGURE 3:**
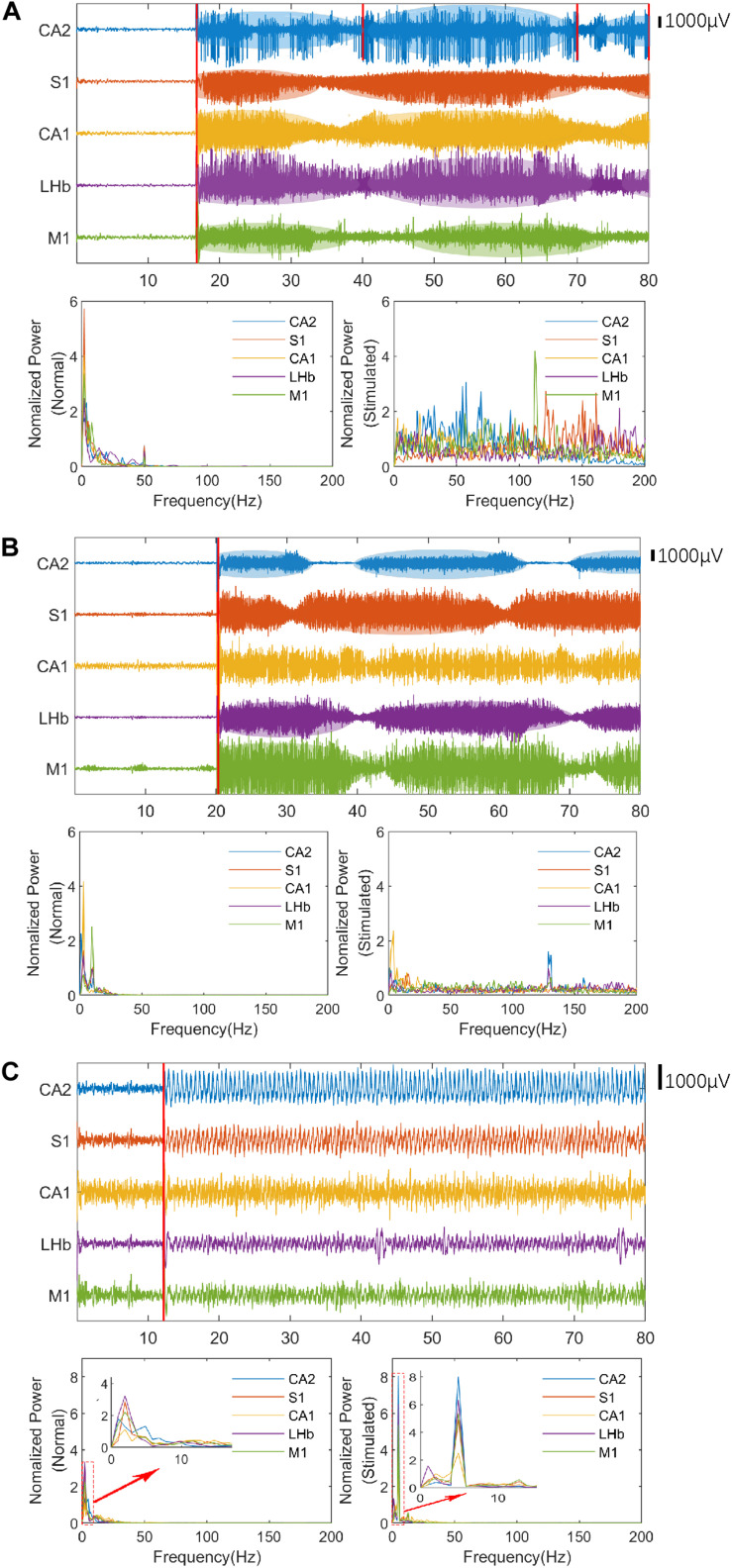
The LFPs and spectral estimation in the rat before (normal state) and after ultrasonic stimulation. The stimulation time of ultrasound is marked with a red line. **(A)** The LFPs and spectral estimation with band-pass filtering (PRF = 500 Hz, *I*_*SPTA*_ = 16.4 mW/cm^2^, 1–140 Hz) and notch filter (50 Hz). **(B)** The LFPs and spectral estimation with band-pass filtering (PRF = 1 kHz, *I*_*SPTA*_ = 20.8 mW/cm^2^, 1–140 Hz) and notch filter (50 Hz). **(C)** The LFPs and spectral estimation with band-pass filtering (PRF = 1.5 kHz, *I*_*SPTA*_ = 8.8 mW/cm^2^, 1–45 Hz). Red rectangles indicate the period expanded in the small window.

Enhanced neural oscillation (the average amplitude of LFPs were 4–10 times higher than normal) with rhythmic patterns appeared in selected nuclei during VLUTS with PRF of 500 and 1 kHz, when the I_*SPTA*_ raised to 12 mW/cm^2^ (Table 1 and Figure 3). Under continuous ultrasonic stimulation, enhanced voltage presented such increase-subside rhythm repeats periodically with very slow frequency, around 0.03–0.05 Hz, which bears some similarity to autorhythmicity. We define these types of oscillations pattern to be auto-rhythm. As shown in Figure 3A, the red line marks one start and end period of CA2 nucleus rhythm. The number of responding nuclei reached maximum when I_*SPTA*_ raised to 16.4 mW/cm^2^ for PRF 500 Hz and 20.8 mW/cm^2^ for PRF 1 kHz (Table 1). And the number of responding nuclei started to decrease while I_*SPTA*_ continued to increase. All such auto-rhythmic oscillations were highlighted with shadows (Figures 3A,B). The average amplitude of LFP showed in Figure 3 was listed in Table 2. Under PRF 1.5 kHz, neural oscillation was moderately increased during stimulation but without the auto-rhythm.

**TABLE 2:** Average Power of LFPs before and after stimulation.

**Units: μ W**	**PRF = 500 Hz**		**PRF = 1 kHz**		**PRF = 1.5 kHz**	
	**Normal**	**Stimulation**	**Normal**	**Stimulation**	**Normal**	**Stimulation**
CA2	0.018	0.769	0.008	0.111	0.014	0.204
S1	0.018	0.503	0.011	0.835	0.021	0.116
CA1	0.020	0.898	0.053	0.436	0.067	0.097
LHb	0.009	0.749	0.006	0.475	0.012	0.047
M1	0.023	0.268	0.025	0.905	0.047	0.057

The LFP signal is a continuous stochastic process. It consists of a series of continuously varying voltages in time, which encodes rich information of neural activities and can be quantitatively characterized in the frequency-domain by spectral estimation. The LFP signals of individual channels before and after the ultrasound stimulation were processed by the multitaper spectral estimation technique as illustrated in Figure 3. Compared to neuronal activity prior to ultrasound stimulation, all five nuclei maintained higher excitability with the auto-rhythmic pattern during VLTUS with PRF 500 Hz, *I*_*SPTA*_ = 16.4 mW/cm^2^ and PRF 1 kHz, *I*_*SPTA*_ = 20.8 mW/cm^2^, and increased high-frequency energy (slow gamma and high gamma) was observed. Intriguingly, the CA1 nuclei didn’t show auto-rhythmic pattern during VLTUS with PRF 1 kHz (Figure 3B). Under PRF 1.5 kHz stimulation, the low frequency delta wave (4 Hz) was dramatically increased (Figure 3C).

Table 1 shows numbers of nuclei which showed significant neuromodulations with auto-rhythmic pattern under different UTS parameters. Under PRF 500 Hz, all five nuclei showed the positive response at *I*_*SPTA*_ = 16.4 mW/cm^2^. Under PRF 1 kHz, four nuclei responded at *I*_*SPTA*_ = 20.8 mW/cm^2^. With increase the AI, the number of responding nuclei started dropping. When I_*SPTA*_ raised to 25.6 mW/cm^2^ only two nuclei under PRF 500 Hz, three nuclei under PRF 1 kHz showed positive response. Under PRF 1.5 kHz, no auto-rhythmic pattern has been observed.

We measured the average power before and after stimulation (10 s each). Results are shown in Table 2. The average power of normal LFPs was within 0.07 μW, but when PRF was 500 Hz and 1 kHz, the average power of LFPs increased by 13–40 times. It can be seen that the transcranial ultrasound induced neurons to open ion channels and discharge. When the PRF is 1.5 kHz, the power slightly increases with obvious group slow wave, but there is no rhythmic pattern. Definitions of ultrasound parameters please see Table 3.

**TABLE 3:** Definitions of ultrasound parameters.

**Parameter**	**Abbreviation**	**Unit**
Fundamental frequency	FF	kHz
Intensity: spatial-peak, pulse-averaged	I_*SPPA*_	W/cm^2^
Intensity: spatial-peak, temporal-averaged	I_*SPTA*_	mW/cm^2^
Pulse length	PL	ms
Pulse repetition frequency	PRF	Hz
Burst duty cycle	BDC	%
Duty cycle	DC	%
Number of cycles per pulse	NC/p	–
Total time	TT	s
Mechanical index	MI	–
Anterior-posterior	AP	
Medial-lateral	ML	
Dorsal-ventral	DV	

**FIGURE 4:**
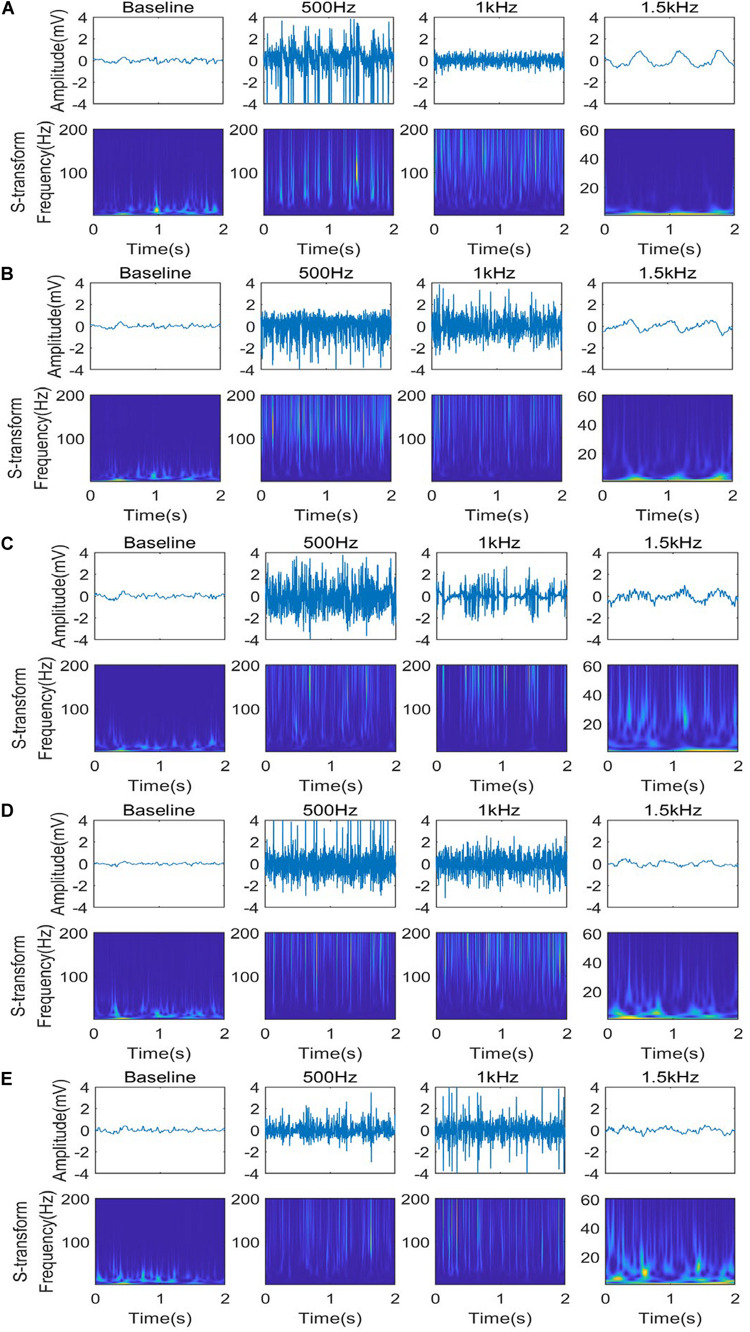
Time-frequency analysis of different stimulation PRF for five nuclei: CA2 **(A)**, SI **(B)**, CA1 **(C)**, LHb **(D)**, and MI **(E)** under different ultrasound stimuli (PRF = 500 Hz, *I*_*SPTA*_ = 16.4 mW/cm^2^, PRF = 1 kHz, *I*_*SPTA*_ = 20.8 mW/cm^2^, PRF = 1.5 kHz, *I*_*SPTA*_ = 8.8 mW/cm^2^).

We utilized the TFA technology to analyze these phenomena further (Figure 4). To avoid high computational complexity, we only analyzed the LFP signal of five nuclei in 1 epoch (2S), starting from 4 s after stimulation to eliminate the noise at the beginning of stimulation. The normal LFP signal energy is mainly concentrated between 2 Hz and 45 Hz. After stimulation, the high frequency component (60–200 Hz) of the LFP is prominent when PRF = 500 Hz and 1 kHz, and the low frequency (1–8 Hz) appear and dominant when PRF = 1.5 kHz. These phenomena are consistent with the results discussed in Figure 3.

### Relative Power of Different Rhythms During TUS With Different PRF

The baseline of the stimulation or inhibition is calculated by 6 s normal LFPs (P0: reference power of normal LFPs). And LFP signals within three epochs (6 s) just 4 s after stimulation (to eliminate abnormal interference of brain at the beginning of stimulation) are used to analyze the relative power. To demonstrate the relative stimulation or inhibition effect of different PRF on each rhythm (compared with normal LFP signal without stimulation), the relative power of LFP signals was divided by the relative power of normal LFP signals, and the logarithm of the result was taken.

**FIGURE 5:**
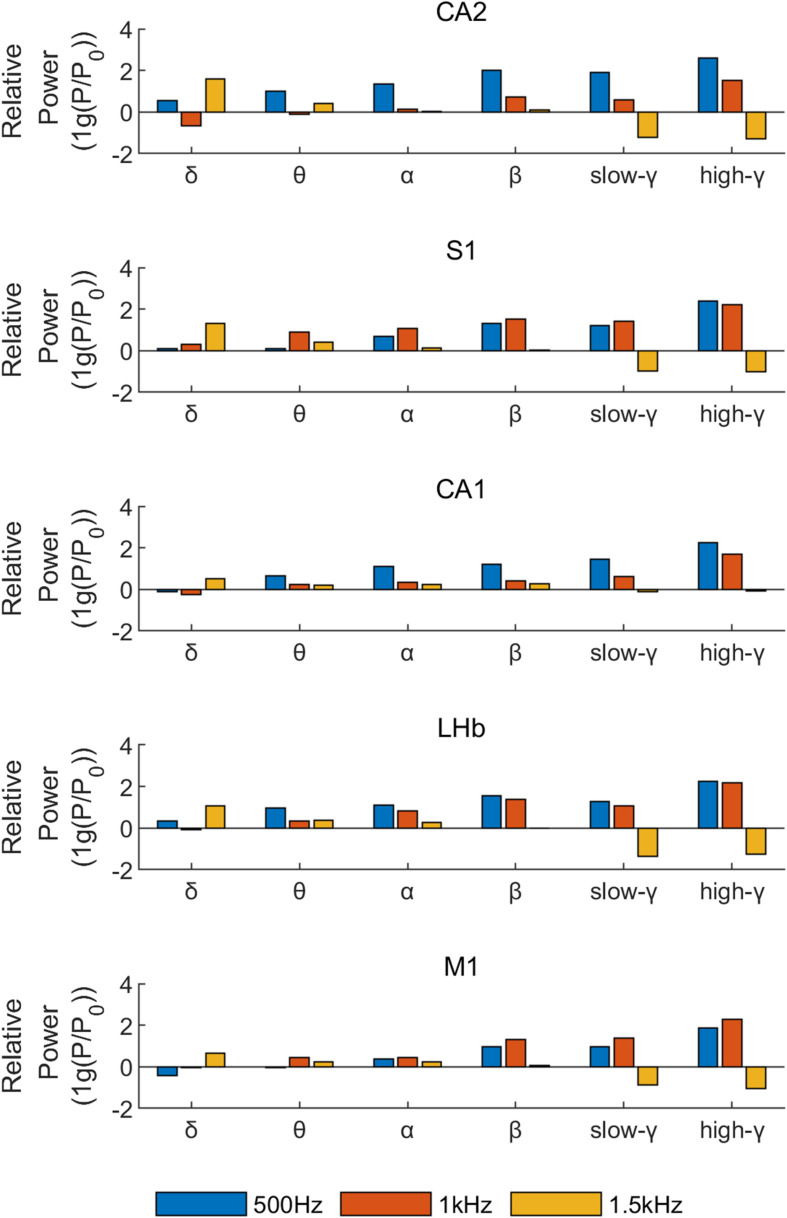
The relative power of different rhythms for five nuclei under different ultrasound stimuli (PRF = 500 Hz, *I*_*SPTA*_ = 16.4 mW/cm^2^, PRF = 1 kHz, *I*_*SPTA*_ = 20.8 mW/cm^2^, PRF = 1.5 kHz, *I*_*SPTA*_ = 8.8 mW/cm^2^).

As we can see from Figure 5 when PRF 500 Hz or PRF 1 kHz the gamma component of the LFP, especially high-gamma component of the LFP (hgLFP), was significantly increased after stimulation, the theta and delta power decreased otherwise. However, when PRF 1.5 kHz high frequency band are suppressed while delta rhythms increased.

**FIGURE 6:**
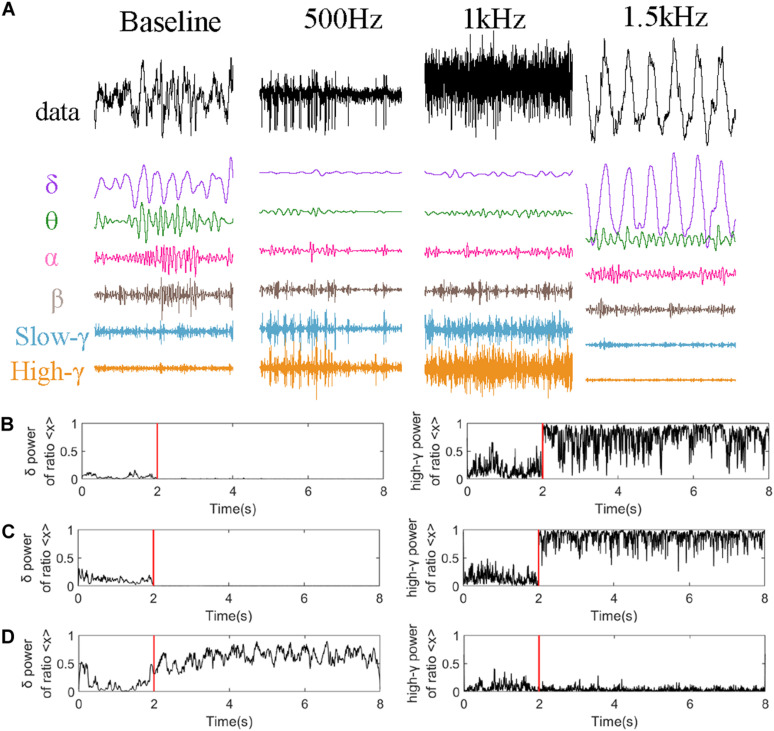
Rhythm analysis of CA2 nucleus. **(A)** Decomposition under different stimulation parameters (PRF = 500 Hz, *I*_*SPTA*_ = 16.4 mW/cm^2^, PRF = 1 kHz, *I*_*SPTA*_ = 20.8 mW/cm^2^, PRF = 1.5 kHz, *I*_*SPTA*_ = 8.8 mW/cm^2^). High-gamma and delta power ratio of the whole signal at **(B)** PRF = 500 Hz, *I*_*SPTA*_ = 16.4 mW/cm^2^, **(C)** PRF = 1 kHz, *I*_*SPTA*_ = 20.8 mW/cm^2^, and **(D)** PRF = 1.5 kHz, *I*_*SPTA*_ = 8.8 mW/cm^2^.

Figure 6 analyzed LFP signal of CA2 nucleus in one epoch (2S) just 4 s after stimulation as an example. Figure 6A represents the decomposition of the LFP signal of CA2 nucleus into different rhythms. And we analyzed the power ration of delta and high-gamma rhythms which is changed obviously in Figure 5 under different stimulation parameters. The high-gamma power ratio (Figures 6B,C) was significantly increased after stimulation, while the theta power suppressed. When PRF 1.5 kHz (Figure 6D) the powers ration of the theta and delta frequency bands were increased obvious, however, while the high-gamma power ratio remains the same.

Here we convincingly prove that the hgLFP is active when PRF 500 Hz and 1 kHz. And the awakening of delta and theta rhythm is a particular phenomenon when PRF 1.5 kHz.

## Discussion

The VLTUS exerted strong neuromodulation effect in our study, leading us to hypothesize that the membrane gating kinetics modulated by VLTUS may induce strong synchronized synaptic currents. In fact, The mechanical interaction between US and neuronal membranes can modify the membrane gating kinetics through the action on mechanosensitive voltage-gated ion channels or neurotransmitter receptors (Sukharev and Corey, 2004; Morris and Juranka, 2007; Tyler et al., 2008). We observed rhythmic high-amplitude LFP activities during low frequency and low power TUS (PRF 500 Hz and 1 kHz, *I*_*SPTA*_ 12–25.6 mW/cm^2^) with high frequency energy. The normal amplitude of the LFP can range from a few microvolts to hundreds of microvolts in the cortex (Goldberg et al., 2004). However, much higher LFP amplitude (>1,000 μV) was observed in this study during VLTUS. LFPs represent the summed voltage fluctuations among a population of cells within a given field and are measured with electrodes. The amplitude of LFP signals is determined by correlated activities of the neurons that receive the inputs at the recording site (Goldberg et al., 2004). It indicates that synchrony of synaptic currents is enhanced during VLTUS. The underlying physiology remains unclear.

### The Influence of PRF Frequency on the Neural Activity

To the best of our knowledge, this is the first report of neurons sensitive to the specific frequency and AI during ultrasound stimulation. And we could find that neurons responding selectively to inputs of a specific frequency.

When PRF 500 Hz or PRF 1 kHz, neurons show prefer at gamma frequencies. The increased power of hgLFP during VLTUS is a kind of neural excitement. The physiological reasons for the elevated power of hgLFP phenomenon are not clear, but previous studies can provide some insights for its source. One potential explanation is that high-frequency (>60 Hz) components of signals represent activation of neuronal populations in the underlying cortex (Crone et al., 2006). Contamination of spike waveforms from nearby cells, either too small or too variable to be detected as single unit spikes (Berens et al., 2008). TUS under PRF 500 Hz or 1 kHz could wake up and activate hgLFP. Recent data recorded from animals and humans have suggested that gamma-frequency activity plays an important role in both attention and long-term memory (Jensen et al., 2007). Such hgLFP with auto-rhythm performance could aid in understanding neuronal processing in complex cognitive functions in future studies.

The slow waves (theta and delta frequency band) appear and occupy when PRF is 1.5 kHz. Theta rhythms are produced by local interactions between hippocampal interneurons and pyramidal cells. There is evidence that mammalian brain display intrinsic resonance with frequency selectivity for electrical stimulation inputs within the theta-range (4–10 Hz) (Hutcheon and Yarom, 2000; Vera et al., 2017). Seidenbecher et al. (2003) have found that synchronization of theta activities in the amygdalohippocampal network represents a neuronal correlate of conditioned fear, apt to improve neuronal communication during memory retrieval. This may be related to the fact that ultrasound causes abnormal neuronal activity, so neuronal resonance of theta and delta rhythms might be waked up, relevant to cognitive operations and learning in the brain.

### The Mechanism of Auto-Rhythmic Pattern

The mechanism of auto-rhythmic pattern of neural oscillation during VLTUS remains unknown but could relate to the refractory period observed in neurons. Refractory periods are caused by the inactivation property of voltage-gated sodium channels and the lag of potassium channels in closing. Voltage-gated sodium channels have two gating mechanisms, the activation mechanism that opens the channel with depolarization, and the inactivation mechanism that closes the channel with repolarization. While the channel is in the inactive state, it will not open in response to depolarizing stimuli. LFP reflects the neural population activity. The synchronization of refractory periods amongst groups of neurons changes population neuronal activity between depolarized and hyperpolarized phases. This may cause that rhythmic pattern. Interestingly, the rhythmic patterns in five nuclei are not identical (Figure 3), which may be due to physiological features of the neuron and the characteristics of the extracellular field in each nucleus.

All five nuclei showed the auto-rhythmic neuromodulation with high frequency energy during the VLTUS (PRF 500 Hz, *I*_*SPTA*_ 16.4 mW/cm^2^**).** All nuclei are in distinct brain networks and located distally, while focus ultrasound stimulation is localized. It’s hard to conclude that all electrical signals were from post synaptic action. Previous studies showed that spatiotemporal fluctuations of the extracellular field may influence *via* purely electrostatic, so-called ephaptic coupling (Jefferys, 1995; Weiss and Faber, 2010). Network oscillations across and within brain areas are critical for brain function (Roach et al., 2018). Coherent oscillations could provide such a mechanism in coordinating the timing of activity and enhancing the functional link between distant areas. Ephaptic coupling may play a big role in this procedure. Our data support those findings and indicate that the ephaptic coupling could widely exist in brain networks, which strongly entrain action potentials (Anastassiou et al., 2011). Ephaptic coupling has been shown to affect population activity during hypersynchronous epileptic discharges (Jefferys, 1995; McCormick and Contreras, 2001). However, how ephaptic coupling alters the functioning of neurons under physiological conditions remains unclear. Our data suggest that VLTUS could be a valid tool in this field.

### Limitation

A larger sample study will help us better evaluate the neuromodulation effect of VLTUS; Only selective PRF and AI have been tested in this pilot study. Need further experiments to prove the mapping relationship between simulation position with the five recording nuclei; The hydrophone can be used to characterize the acoustic field with the focal length and focal length of the transducer measured accurately.

## Conclusion

In summary, VLTUS shows a significant neuromodulation effect. Both the amplitude and frequency power of LFPs were altered during specific stimulation parameters. VLTUS could be a valid tool to study neurons’ physiological conditions, including ephaptic coupling.

## Data Availability Statement

The original contributions presented in the study are included in the article/supplementary material, further inquiries can be directed to the corresponding author/s.

## Ethics Statement

The animal study was reviewed and approved by the Laboratory Animal Ethical and Welfare Committee of Shandong University Cheeloo College of Medicine.

## Author Contributions

YLiu: acquisition of data, analysis and interpretation of data, drafting, and revising the article. YZ, WZ, and YLi: design of the experiments, analysis and interpretation of data, and revising the article. GW, CC, GZ, and ET: analyzed the data and wrote the manuscript. All authors contributed to the article and approved the submitted version.

## Conflict of Interest

The authors declare that the research was conducted in the absence of any commercial or financial relationships that could be construed as a potential conflict of interest.

## Appendix

### Acoustic Intensity Calculation

Using the radiation force method, we could calculate the acoustic power. The pressure of radiation (*P*_τ_) is equal to the energy density (E):

(3) $$P_{\tau }=E$$

We use a precision balance and a radiation force target that is perfectly absorptive. The conversion from force to ultrasonic power can be accomplished by using a simplified equation:

(4) P=w×14.56

where w is the ultrasonic force in grams and *p* is the ultrasonic power in watts.

Power intensity is rated as Spatial Peak Temporal Average (I_*SPTA*_) and Spatial Peak Pulse Average (I_*SPPA*_). I_*SPPA*_ is defined as

(5) $$I_{S\,P\,P\,A}=\frac{\text{P}}{\text{S}}$$

where *S* is the cross-sectional area of the given transducer. And I_*SPTA*_ is defined as:

(6) $$I_{S\,P\,T\,A}=I_{S\,P\,P\,A}\cdot \left(\mathrm{DC}\right)$$

where DC is the duty cycle.

Brain and tissues were assumed to have the sound speed and density of water. Moreover, in order to get the acoustic intensity transmitted into the brain, the absorption and refraction characteristics in the skull was considered. The acoustic intensity (I) after passing through skull can be estimated as (O’Brien, 2007)

(7) $$I=I_{0}\,{\text{e}}^{-\alpha \,\text{l}}$$

Where I_0_ is the initial intensity, α is the attenuation coefficient [3.5 dB/cm in rat’s skull at 0.5 MHz (Culjat et al., 2010)], and l is the thickness of skull of rat [about 0.5 mm (Younan et al., 2013)].
